# Experimental and Numerical Simulation of Reinforced Concrete-Filled Square GFRP Tubular Columns under Axial Compression

**DOI:** 10.3390/ma17112595

**Published:** 2024-05-28

**Authors:** Chenggui Jing, Lin Zhao, Tong Wu, Weizhao Li

**Affiliations:** School of Civil Engineering and Architecture, Guangxi University of Science and Technology, Liuzhou 545006, China; jingchg06@163.com (C.J.); 19982030205@163.com (L.Z.); 18354883098@163.com (T.W.)

**Keywords:** GFRP square tube, spiral reinforcement, confined concrete, high-strength steel wire, bearing capacity

## Abstract

To investigate the axial compressive behavior of reinforced concrete-filled square glass-fiber-reinforced polymer(GFRP) tubular (RCFSGT) columns, 17 specimens were designed with variations in GFRP tube wall thickness, spiral reinforcement yield strength, and spiral reinforcement ratio. A detailed model was developed using the finite element software ABAQUS, enabling in-depth mechanistic analysis and expanded parameter studies. The results indicate that the failure types of the specimens are all manifested as GFRP square tube cracking, and the core concrete is subjected to crushing or shear failure. The inclusion of a reinforcement cage can significantly enhance the load-bearing capacity and ductility of the specimen. Furthermore, as the yield strength and reinforcement ratio of the spiral reinforcement increase, so does the load-bearing capacity of the specimen. The finite element simulation results align well with the experimental findings. As the wall thickness of the GFRP square tube increases from 2 mm to 6 mm, the load-bearing capacity improves by approximately 19.69%. With the yield strength of the spiral reinforcement rising from 200 MPa to 400 MPa, the specimen’s load-bearing capacity shows an increase of approximately 7.55%. However, as its yield strength continues to increase, there is minimal change in the load-bearing capacity. When the stirrup ratio of spiral reinforcement rises from 0.33% to 2.26%, the specimen’s load-bearing capacity experiences an increase of approximately 56.90%.

## 1. Introduction

Fiber-reinforced polymer (FRP) exhibits advantages such as being lightweight and highly strong (with a density approximately one-quarter that of steel and a tensile strength that can exceed 10 times that of conventional carbon structural steel), excellent corrosion and durability resistance, and strong designability. It is extensively utilized in civil engineering [1]. In engineering practice, common applications of FRP include the reinforcement of existing structures with FRP cloth, the substitution of conventional steel bars with FRP bars, and FRP tube concrete [2,3,4].

Mirmiran [5] was the first to suggest casting concrete into FRP tubes, thereby creating a FRP tube–concrete composite structure. Not only do FRP tubes provide excellent support for the concrete, but they also act as templates, enhancing construction speed and ensuring durability. Numerous scholars have conducted extensive research on the mechanical properties of FRP-encased concrete. Liu [6], Togay Ozbakkaloglu [7,8], Yan [9], and Li [10] et al. conducted axial compression tests on concrete confined by FRP tubes (CFSGT) of different types and different cross-sections. The test results showed that the constraint principle of FRP tubes to concrete was the same as that of steel tubes, and both of them improved the axial compression strength and ultimate strain of concrete by lateral constraint. Dang [11], Tian [12], and Liao [13] et al. conducted axial and eccentric compression tests on cement-based composite materials such as ECC and UHPC, confined by FRP tubes. This research revealed that FRP-confined ECC specimens exhibit similar axial compressive hardening characteristics to FRP-confined ordinary concrete specimens on the stress–strain curve. The self-confinement effect of ECC and the confinement of FRP have significant effects on the stress–strain response and failure mode. The results of the eccentric compression performance of FRP-confined UHPC indicate that all specimens except for the large eccentricity specimens exhibit strain-hardening behavior. With the increase in eccentricity, the bearing capacity and deformation capacity significantly decrease, and the thickness of the tube only shows a significant impact in concentric or small eccentricity cases. YIN et al. [14] integrated steel ribs into the concrete of GFRP tubes and examined the force transmission mechanism in GFRP-tube-reinforced concrete composite short columns using experiments and finite element analysis. They observed that the restraint provided by the GFRP tubes on the concrete occurred slightly later than that by the steel ribs, exhibiting a certain degree of delay. XEI [15] and ZHANG [16] et al. conducted experimental research on FRP tube concrete with built-in profiles. The findings indicate that the ultimate bearing capacity of FRP tube concrete rises with the increase in FRP tube thickness, for both built-in steel and FRP profiles. Nevertheless, enhancing the thickness of the FRP tube does not significantly affect the ductility and stiffness of the specimens. FENG et al. [17,18,19,20,21,22,23] conducted an experimental study on concrete columns with double-tube (external FRP tube, internal steel tube or external steel tube, internal FRP tube) confinements. The test results show that the new type of composite column exhibits excellent mechanical properties and good deformability. HE et al. [24] conducted experimental research on the axial compression performance of CFSGT columns after freeze–thaw cycles. The test results showed that the ultimate bearing capacity and initial stiffness of CFSGT columns decreased significantly with the increase in freeze–thaw cycles. GUO et al. [25] investigated the axial compressive mechanical behavior of CFSGT columns after exposure to high temperatures. The research revealed that as temperatures rose, both the specimens’ ultimate bearing capacity and initial stiffness declined notably. Specifically, the ultimate bearing capacity dropped by approximately 37% when temperatures exceeded 200 °C.

FRP is a brittle material, and the failure mode of FRP-confined concrete is a brittle failure. Spiral-reinforced concrete is considered a method of constraint with high efficiency, steel conservation, and ease of industrial production [26]. Currently, the production process for GFRP materials is well established, and the cost is lower compared to CFRP. Therefore, the integration of GFRP tubes and spiral reinforcement to enhance concrete not only addresses the issue of brittle failure in GFRP-confined concrete but also mitigates the severe corrosion problem of steel in traditional reinforced concrete structures. The enhanced durability and seismic performance of the component arise from the composite reinforcement of FRP tubes and steel bars. Employing this component can prolong the lifespan of the structure and contribute to a reduction in carbon emissions throughout the entire construction process. However, to date, there has been limited research on RCFSGT columns. Consequently, this study conducted 4 sets of experiments with 17 specimens under axial compression, revealing the influence pattern of RCFSGT columns on axial compression bearing capacity. Building on these experimental findings, a finite element model was developed for mechanistic analysis and parameter expansion research. These findings contribute to advancing the theoretical research and practical engineering applications of RCFSGT structures.

## 2. Experimental Investigation

### 2.1. Test Specimens

This study designed 17 column specimens, comprising 12 specimens of RCFSGT, 2 specimens of GFRP square tube, 1 specimen of CFSGT, and 2 specimens of reinforced concrete. The specimens measure 300 mm in height and 100 mm in width (including wall thickness). The GFRP square tube used in the test has wall thicknesses of 3 mm and 6 mm. The spiral reinforcement specifications include galvanized iron wire and high-strength steel wire, with three different spiral reinforcement spacings of 22 mm, 15 mm, and 10 mm. The longitudinal reinforcement is made of galvanized iron wire. Both the spiral reinforcement and longitudinal reinforcement have a diameter of 3 mm, and the design strength of the concrete is C40. The main parameters of the test piece are presented in Table 1. In Table 1, *t* represents the wall thickness of the FRP square tube, *S* represents the spacing of spiral reinforcement, *f*_hy_ represents the yield strength of spiral reinforcement, and *n* represents the number of longitudinal bars.

**Figure 1:**
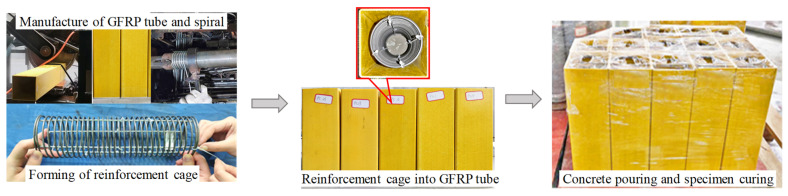
The production process for specimens.

The main fabrication processes for the test specimen include: (1) Cutting of the GFRP tube and processing and shaping of the spiral reinforcement. (2) Sealing the GFRP tube with a circular wooden board to prevent slurry leakage during concrete pouring. (3) Assembling the reinforcement cage and inserting it into the GFRP tube. (4) Specimen fixation and concrete pouring. The fabrication process is depicted in Figure 1.

### 2.2. Materials

**Figure 2:**
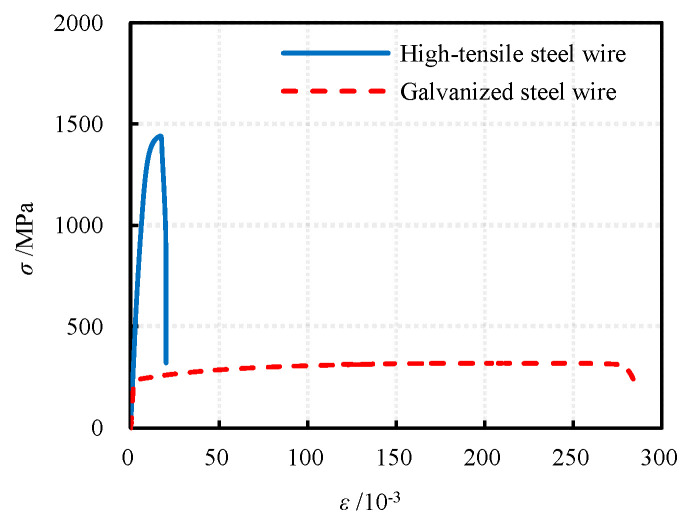
Stress–strain curve of steel.

During the pouring of concrete, 6 concrete cube test blocks measuring 100 mm × 100 mm × 100 mm were set aside. The curing conditions are identical to those for the specimens. The compressive strength of the concrete was determined, with an average value of 57.6 MPa. The tensile properties of GFRP material were determined according to GBT1447-2005 [27]. Three transverse and three axial tensile specimens were made, respectively, and the loading speed was 2 mm/min. During the loading process, the plies orthogonal to the tensile load direction will be damaged first, followed by the gradual tearing of the ±45° plies. Conversely, the plies parallel to the stress direction have the most significant impact on the tensile properties of the profile. The compressive properties of GFRP materials were tested in accordance with GBT1448-2005 [28], with a loading speed of 2 mm/min. The main plies of the axial compression specimens were aligned with the direction of force application. Under compressive loads, the varying degrees of progressive damage among the plies result in coordinated deformation, causing the interlayer interfaces with weaker bonding to tear away, leading to the vertical splitting of the entire profile after delamination. In the transverse compression specimen, the primary plies are oriented perpendicular to the direction of applied stress, while the secondary plies undergo compressive buckling under compressive loads, ultimately resulting in mid-specimen cracking and fracture. The mechanical properties of the GFRP tube material, as measured, are presented in Table 2. The tensile properties of steel are determined in accordance with GB/T 228.1-2021 [29]. The measured stress–strain curve is depicted in Figure 2, and the tensile mechanical properties are presented in Table 3.

### 2.3. Test Setup and Loading Program

**Figure 3:**
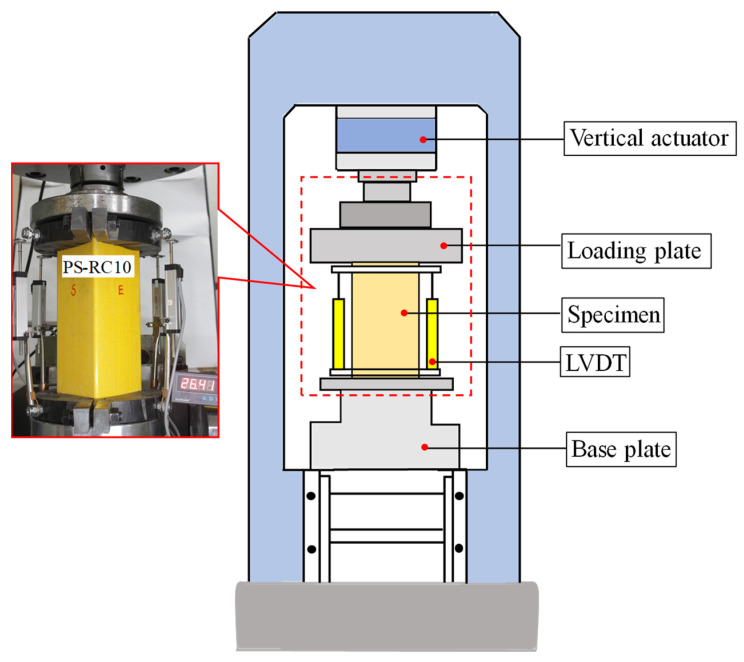
Diagram of test specimen and loading device.

The RMT-301 rock and concrete mechanics testing machine was utilized to subject specimens to monotonic static loading. The loading system was controlled by displacement, with a loading rate of 0.02 mm/s. To avoid local stress concentration in the specimen during loading, a pair of clamps with a protective width of 15 mm are set at both ends of the specimen. The axial load on the test piece is determined using the press’s built-in acquisition system. Displacements are measured using four displacement meters positioned around the specimen, with the data being captured by the Donghua static strain test system. The fixture and loading apparatus are depicted in Figure 3.

## 3. Experimental Results and Analysis

### 3.1. General Behavior and Failure Modes

**Figure 4:**
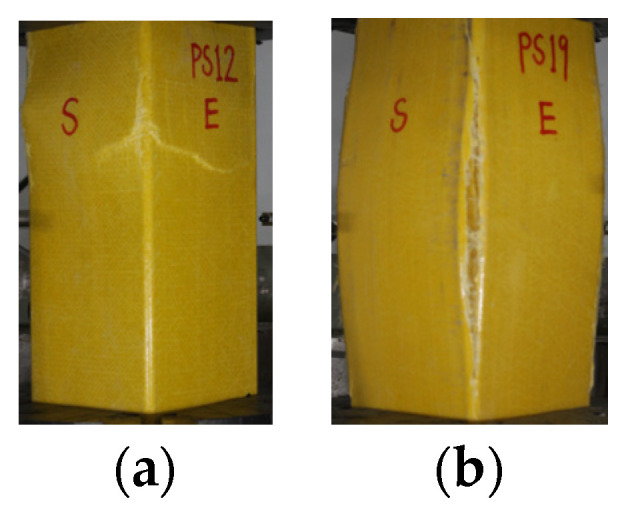
Apparent failure characteristics of specimens. (**a**) Surface oblique cracks. (**b**) Corner fracture due to expansion.

The failure process of concrete-filled thin-walled GFRP tubular columns involves: (1) an elastic phase, wherein there are no evident changes in the specimen appearance, with the sounds of GFRP fiber tension and resin fracture being heard. (2) an elastic–plastic phase, when the axial load increases to approximately 90% of the peak load and major cracks emerge at the corners of the GFRP tube surface, accompanied by a clear cracking sound. (3) a descending phase, when the peak load is reached, longitudinal cracks emerge along the four corners of the GFRP tube, the load drops rapidly, the GFRP tube ruptures transversely, leading to specimen failure, and the loading ceases. The initial two stages of the failure process in the thin-walled RCFSGT column resemble those in the CFSGT column. In the descending phase, once the specimen reaches its peak load, the rapid development of cracks occurs at the GFRP tube’s corners, leading to the emergence of inclined cracks and the formation of pleated patterns, as depicted in Figure 4a. During the late loading phase, there is a sudden decrease in load followed by the distinct sound of steel rebar breaking. As a result, the specimen fails, and the loading ceases.

In the elastic and elastic–plastic loading phases, the thick-walled RCFSGT column exhibits no significant changes in appearance. However, in the descending phase, upon reaching the peak load, the corner of the specimen undergoes expansion and fracturing, leading to a sudden load reduction, as depicted in Figure 4b. This phenomenon is consistent with the failure mode of specimens with wall thicknesses of 5 mm and 6 mm as mentioned in GUO [30], which is mainly due to the debonding at the interface between the internal concrete and the GFRP square tube, and the tearing and penetration at the corner of the GFRP square tube.

**Figure 5:**
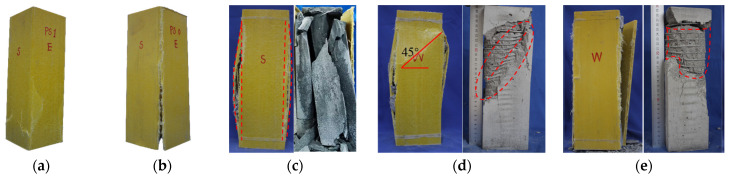
The appearance and internal failure mode of the specimen. (**a**) Thin-walled GFRP tube. (**b**) Thick-walled GFRP tube. (**c**) Thin-walled CFSGT column. (**d**) Thin-walled RCFSGT column. (**e**) Thick-walled RCFSGT column.

The typical failure mode of the specimens is depicted in Figure 5. In general, the specimens fail due to the instability, fracture of GFRP tubes, or fracture of spiral reinforcement, yet there are variations in the failure modes. The failure modes of GFRP hollow tubes with different wall thicknesses vary. Figure 5a,b indicate that thin-walled GFRP hollow tubes fail through instability, while GFRP square tubes undergo buckling. Thick-walled GFRP tubes exhibit global failure, with the corners of the GFRP square tubes fracturing. Figure 5c indicates that the concrete column in the thin-walled GFRP tube undergoes fracturing, with longitudinal cracks emerging at the four corners of the GFRP square tube. The internal concrete is cleaved and fragmented into discrete small columns. This is because, after the compression failure of the GFRP square tube, the constraint effect on the internal concrete is greatly reduced, resulting in the uniaxial compression state of the concrete. The final failure mode is similar to that of the concrete prism specimen. Figure 5d,e indicate that the thin-walled RCFSGT column undergoes shear failure (approximately 45°). Obvious diagonal cracks emerge on the GFRP square tube’s surface. Upon stripping away the GFRP square tube, the concrete interface between the GFRP square tube and the spiral reinforcement exhibits significant disruption, particularly at locations corresponding to the diagonal cracks in the GFRP square tube. On the shear plane, the spiral reinforcement frequently exhibits signs of necking and breakage. The thick-walled RCFSGT column, characterized by spiral reinforcement, experiences a swelling failure of the GFRP square tube, resulting in a longitudinal crack at the corner of the GFRP square tube, which runs through the entire tube along the direction of the column’s height.

### 3.2. Axial Load–Axial Displacement Curve

**Figure 6:**
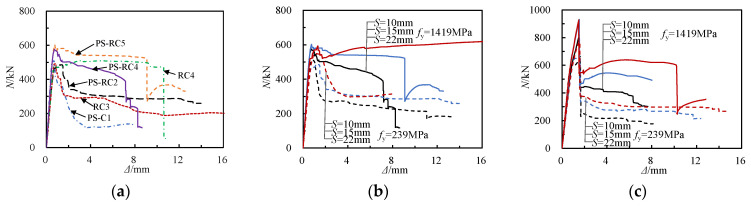
Axial load–axial displacement curve. (**a**) Different combination forms of specimens. (**b**) Thin-walled GFRP-tube-reinforced concrete column with spiral reinforcement. (**c**) Thick-walled GFRP-reinforced concrete column with spiral reinforcement.

The axial load–axial displacement (*N*-Δ) curve of the RCFSGT column with spiral reinforcement is shown in Figure 6. Figure 6a shows that the RCFSGT column PS-RC2 specimen exhibits a modest increase in bearing capacity compared to the spiral-reinforced concrete column RC3 specimen, with a notably slower decline in the curve. This trend is more pronounced when using high-strength spiral reinforcement. This suggests that incorporating GFRP square tubes into spiral-reinforced concrete specimens not only ensures durable maintenance in harsh environments and reduces carbon emissions of the structure but also enhances the specimens’ axial compressive mechanical properties. From the analysis of Figure 6b, the *N*-Δ curve of the thin-walled RCFSGT column with spiral reinforcement exhibits a linear increase during the elastic phase. When the strength of spiral reinforcement increases from 239 MPa to 1419 MPa, the load decrease after the peak is significantly reduced. Moreover, as the spiral reinforcement spacing becomes denser, the ductility of the specimen improves. Figure 6c indicates that during the elastic phase of the *N*-Δ curve, the thick-walled RCFSGT column also exhibits a linear increase. Upon reaching the peak load, there is a sudden decrease in the load on the specimens. This sudden load decrease is primarily attributed to the brittleness of the GFRP square tube, leading to bulging failure. When the strength of the spiral reinforcement is increased from 239 MPa to 1419 MPa, there is a small increase in the bearing capacity of the specimen, but it has minimal impact on ductility. Meanwhile, high-strength steel wires are more prone to brittle fracture, which corresponds to the sudden drop in the *N*-Δ curve in the later stage.

### 3.3. Analysis of Influence Parameters

This study explores the impact of three factors—the wall thickness of GFRP tubes, the yield strength of spiral reinforcement, and the stirrup ratio of spiral reinforcement on the axial compressive mechanical properties of RCFSGT columns, using the ultimate bearing capacity and axial compression ductility (*μ*) as metrics. The ductility coefficient is defined as *μ* [25] = ∆_u_/∆_y_, where ∆_u_ represents the deformation at the limit point and ∆_y_ denotes the deformation at the yield point. The limit point is defined as the point where the *N*-∆ curve drops to 85% of the peak load, and the yield point is determined using the equal energy method [25]. The peak load (ultimate bearing capacity under axial compression), yield displacement, and ultimate displacement [25] of each specimen are extracted from the *N*-∆ curve, as shown in Table 1.

#### 3.3.1. The Influence of the Wall Thickness of GFRP Tube

**Figure 7:**
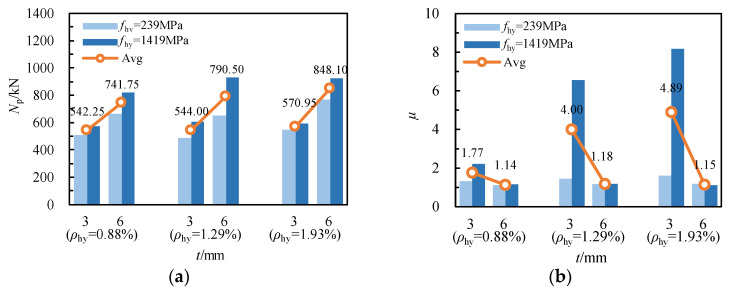
The influence of GFRP tube wall thickness: (**a**) ultimate bearing capacity; (**b**) ductility.

Figure 7 illustrates the impact of the wall thickness of GFRP square tubes on the specimen’s ultimate bearing capacity and ductility. As shown in Figure 7a, with an increase in the wall thickness of the GFRP square tube from 3 mm to 6 mm, and under a stirrup ratio of 0.88% for spiral reinforcement, the average ultimate bearing capacity of specimens equipped with galvanized iron wires and high-strength steel wires rose from 542.30 kN to 741.80 kN, an increase of 36.78%. With a stirrup ratio of spiral reinforcement at 1.29%, the average ultimate bearing capacity rose from 544.00 kN to 790.50 kN, an increase of 45.31%. Similarly, at *ρ*_hy_ = 1.93%, the average ultimate bearing capacity increased from 571.01 kN to 848.10 kN, an increase of 48.51%. The results indicate that increasing the wall thickness of GFRP square tubes significantly enhances the load-bearing capacity of RCFSGT columns. However, as evident from Figure 7b, with the increase in wall thickness from 3 mm to 6 mm for specimens made of galvanized steel wires, the ductility coefficient decreases from 1.32, 1.45, and 1.61 to 1.11, 1.17, and 1.18, resulting in a decrease in their deformability. For high-strength steel wire specimens, the ductility coefficient decreases notably when the wall thickness of GFRP square tubes increases from 3 mm to 6 mm; specifically, When the reinforcement ratios were 0.88%, 1.29%, and 1.93%, the ductility coefficients of the specimens decreased from 2.21, 6.55, and 8.17 to 1.16, 1.18, and 1.11, respectively, with a decrease of about 95.05%, 455.08%, and 636.04%. Notably, the ductility coefficient of GFRP square tube specimens remains at a consistent level of 1.15 when the wall thickness is 6 mm. The wall thickness of GFRP tubes is a pivotal determinant of the ductility of RCFSGT columns. Given the pronounced brittleness of GFRP tubes, this factor significantly impacts their deformability.

#### 3.3.2. The Influence of Spiral Reinforcement on Yield Strength

**Figure 8:**
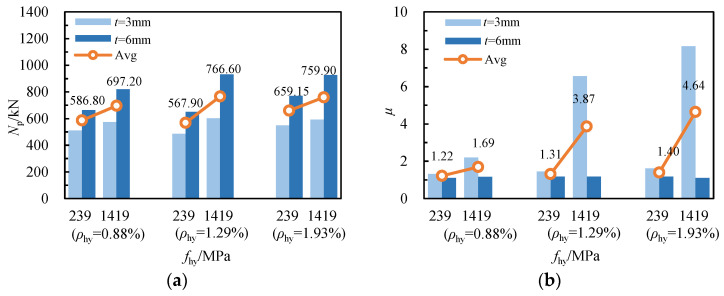
The influence of spiral reinforcement’s yield strength: (**a**) ultimate bearing capacity; (**b**) ductility.

Figure 8a illustrates the influence of spiral reinforcement yield strength on the axial compressive bearing capacity of RCFSGT columns. The yield strength of spiral reinforcement increases from 239 MPa to 1419 MPa for *ρ*_hy_ = 0.88%, *ρ*_hy_ = 1.29% and *ρ*_hy_ = 1.93%, respectively. Consequently, the average bearing capacity of specimens is enhanced by 18.8%, 35.00%, and 15.29%, respectively. This indicates that enhancing the yield strength of spiral reinforcement can significantly improve the bearing capacity of RCFSGT columns within a certain range of stirrup ratio. With wall thicknesses of 3 mm and 6 mm for the GFRP tubes, the yield strength of the spiral reinforcement rose from 239 MPa to 1419 MPa. Consequently, the average ultimate bearing capacities of the specimens increased by 14.73% and 28.41%, respectively. This indicates that enhancing the strength of the spiral reinforcement significantly improves the bearing capacity of specimens, regardless of their tube thickness, be it thin-walled or thick-walled GFRP square tubes.

The influence of the yield strength of spiral reinforcement on the ductility of RCFSGT columns with spiral reinforcement varies. Figure 8b shows specimens with stirrup ratios of 0.88%, 1.29%, and 1.93% for spiral reinforcement, where the yield strength of spiral reinforcement increased from 239 MPa to 1419 MPa, and the average ductility coefficient increased from 1.22, 1.31, and 1.40 to 1.69, 3.87 and 4.64, respectively, representing increases of 38.51%, 195.43%, and 231.42%. When the spiral bars are closer together, the enhancement in the yield strength of spiral bars has a more pronounced impact on the ductility of specimens.

#### 3.3.3. The Influence of Stirrup Ratio of Spiral Reinforcement

**Figure 9:**
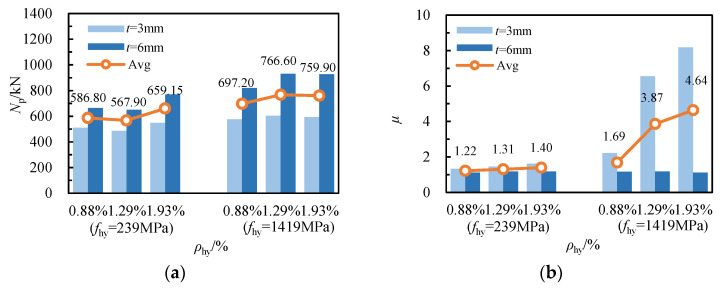
The influence of stirrup ratio: (**a**) ultimate bearing capacity; (**b**) ductility.

Figure 9 compares the ultimate bearing capacity and ductility of specimens with varying stirrup ratios for spiral reinforcement. As shown in Figure 9a, increasing the stirrup ratio for spiral reinforcement from 0.88% to 1.29% reduces the average ultimate bearing capacity of galvanized iron wire specimens from 586.80 kN to 567.90 kN, while increasing it for high-strength steel wire specimens from 697.20 kN to 766.60 kN, an increase of 10.00%. This suggests that when the spiral reinforcement is made of galvanized iron wire and the stirrup ratio is low, the stirrup ratio for spiral reinforcement has minimal impact on the ultimate bearing capacity of RCFSGT columns. When the stirrup ratio of spiral reinforcement is increased from 0.88% to 1.93%, the ultimate bearing capacity of galvanized steel wire specimens rises from 586.8 kN to 659.15 kN, an increase of 12.30%, and the average ultimate bearing capacity of high-strength steel wire specimens increases from 697.20 kN to 759.90 kN, an increase of 9.02%. When the stirrup ratio of spiral reinforcement exceeds 0.88%, increasing it can effectively enhance the ultimate bearing capacity of RCFSGT columns for both galvanized steel wire specimens and high-strength steel wire specimens. As shown in Figure 9b, with the increase in the reinforcement ratio from 0.88% to 1.93% for high-strength steel wire specimens, the average ductility coefficient rises from 1.69 to 4.64, an increase of 174.63%. The influence of the stirrup ratio on the axial compression ductility of specimens is evident. With the stirrup ratio increasing, the specimen’s deformation ductility is enhanced.

## 4. Finite Element Modeling

### 4.1. FE Model Details

**Figure 10:**
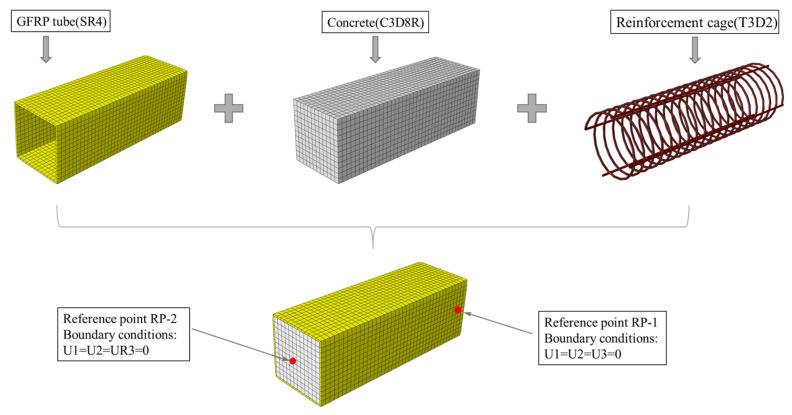
FE modeling for GFRP square-tube-reinforced concrete column with spiral reinforcement.

To further investigate the mechanical behavior of RCFSGT columns with spiral reinforcement under axial compression, a finite element model was established using ABAQUS software 2019. The model comprises a GFRP square tube, concrete, and reinforcement cage. For concrete, the C3D8R solid element is adopted; for spiral and longitudinal reinforcement, the T3D2 truss element is selected; and for the FRP tube, the conventional shell element is used. To ensure computational accuracy, nine integration points are chosen along the thickness direction of the shell element [29]. The grid size is set at 1/10 of the specimen’s cross-sectional size. To prevent stress concentration during loading, two reference points are established at the centroid of the specimen section, with boundary conditions as depicted in Figure 10. The Coulomb friction model is applied to simulate the interfacial shear stress between the square steel tube and concrete; assuming an ideal bond between the reinforcement and concrete, the reinforcement cage is “embedded” within the concrete.

### 4.2. Material Modeling

The constitutive relationship of core concrete is described by the plastic damage model [31,32], and the tensile softening behavior is described by the stress–fracture energy relationship (GFI). The expression for its uniaxial compressive stress–strain relationship is as follows:

(1) $$y=\left\{\begin{array}{c} 2x-x^{2}\left(x\leq 1\right)\\ \frac{x}{\beta_{o}{\left(x-1\right)}^{\eta }+x}\left(x\leq 1\right) \end{array}\right.$$

where *x* = *ε*/*ε*_o_, *y* = *σ*/*σ*_o_; *σ*_o_ and *ε*_o_ is peak stress and peak strain, respectively, *σ*_o_ = *f*_c_^′^, *ε*_o_ = *ε*_c_ + 800*ξ*^0.2^ × 10^−6^, *ε*_c_ = (1300 + 12.5*f*_c_^′^) × 10^−6^, *η* = 1.6 + 1.5/*x*; *β*_o_ is the falling section parameter, *β*_o_ = (*f*_c_^′^)0.1/(1.2(1 + *ξ*)0.5), *f*_c_^′^ is the compressive strength of concrete cylinder, *ξ* is the constraint coefficient effect.

The constitutive relationship for both longitudinal and spiral reinforcement is represented by a double-broken line model [30]. The FRP tube is composed of an elastic material, with its material direction being divided into two: one along the fiber direction and the other perpendicular to the fiber direction. For the stress–strain model, elastic parameters, and damage model, please refer to reference [25].

### 4.3. Verification of Finite Element Models

**Figure 11:**
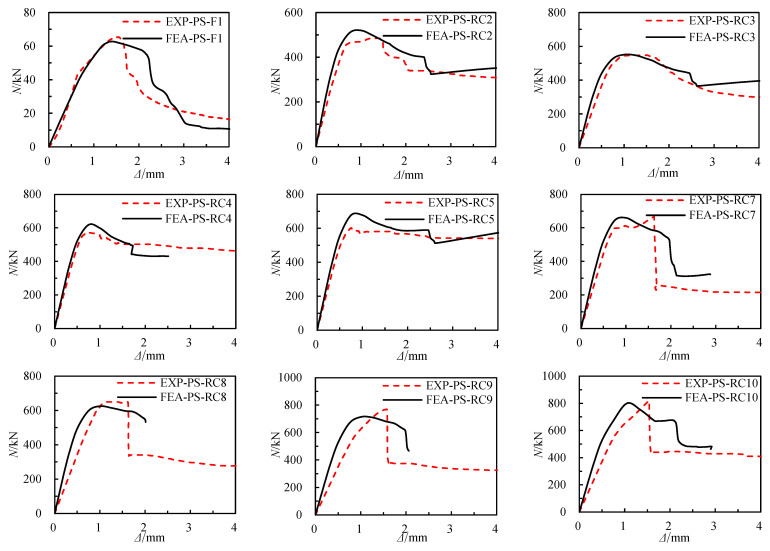
Axial load–axial displacement curves of each component of typical specimens.

Figure 11 compares the axial load–axial displacement responses predicted by the finite element model with typical test results. The *N*-Δ curves of the test piece’s finite element model (FEA) and the test are essentially consistent, despite slight jitter in the test curve due to unstable loading during the test. From Table 4 The average ratio of experimental to finite element-calculated bending bearing capacities is 1.01, with a Standard deviation of 0.09. Therefore, the finite element model can be seen to faithfully simulate the axial compression behavior of RCFSGT columns.

## 5. Stress Mechanism Analysis

**Figure 12:**
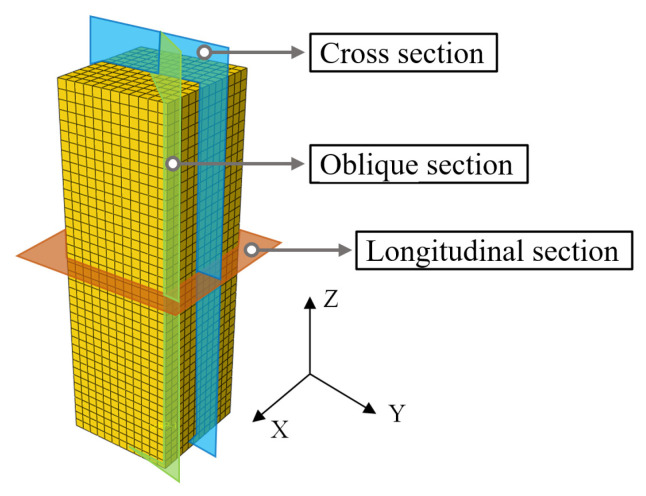
Schematic diagram of section position.

To investigate the influence of spiral reinforcement on the axial compression performance of specimens, the failure modes and strain distribution of GFRP square tube, concrete, and spiral reinforcement were analyzed by taking specimens PS-C1 and PS-RC2 as examples. The position profile of the test piece is shown in Figure 12.

### 5.1. Failure Mode

#### 5.1.1. Failure Mode of GFRP Square Tube

**Figure 13:**
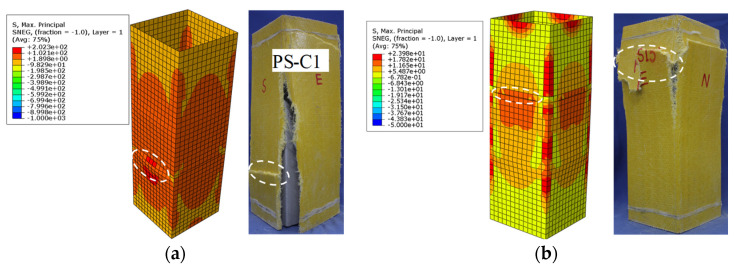
Comparison of failure modes of GFRP tube. (**a**) PS-C1. (**b**) PS-RC2.

Figure 13 compares the GFRP square tube simulations with the observed failure modes of specimens PS-C1 and PS-RC2. The results indicate a strong alignment between the simulations and the experimental observations for the GFRP square tubes of specimens PS-C1 and PS-RC2. Figure 13 indicates that the GFRP tube of test piece PS-C1 fractured in the lower midsection. The stress distribution in the corner of the FRP tube, as revealed by the stress nephogram, is minimal and increases towards the edges. Conversely, for PS-RC2 specimens with spiral reinforcement, the GFRP tube fractured in the midsection, with high corner stress. This suggests that spiral reinforcement effectively confines the core concrete, resulting in stress concentration at the corners of the GFRP square tube.

#### 5.1.2. Concrete Damage under Compression

**Figure 14:**
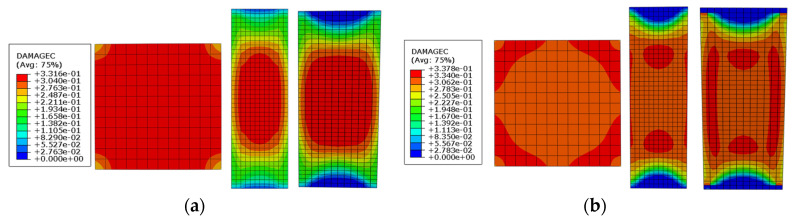
Cloud chart of concrete compression damage corresponding to peak load. (**a**) PS-C1. (**b**) PS-RC2.

Figure 14 presents the concrete damage nephogram of test specimens PS-C1 and PS-RC2, observed in transverse, longitudinal, and oblique sections. The damage in specimen PS-C1 is uniformly distributed across the cross-section. In contrast, the damage in specimen PS-RC2 is primarily concentrated around the cross-section, with less damage in the areas surrounded by spiral reinforcement. This suggests that installing spiral reinforcement within concrete filled GFRP tube can effectively reduce the damage to the core concrete. According to the damage nephogram of the longitudinal section and oblique section, the concrete damage of specimen PS-C1 is concentrated in the middle of the specimen, while the concrete damage of specimen PS-RC2 is evenly distributed in the whole section, and the middle stress is lower than that of specimen PS-RC2, which shows that the spiral reinforcement has good restraint effect and can avoid the concentrated damage of concrete in the middle.

#### 5.1.3. Failure Mode of Spiral Reinforcement

**Figure 15:**
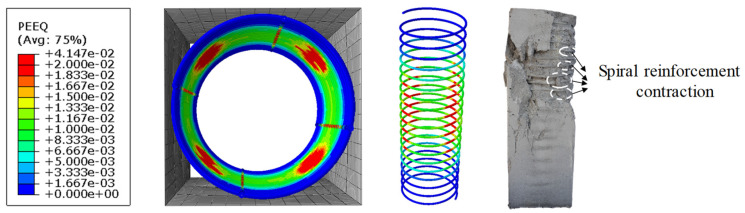
Failure mode of spiral reinforcement.

Figure 15 compares the damage pattern of the spiral reinforcement in specimen PS-RC2 with its final failure mode. As shown in Figure 15, the stress at the corners of the spiral reinforcement is higher than elsewhere, correlating with the necking deformation observed in its central section.

### 5.2. Internal Force Analysis

**Figure 16:**
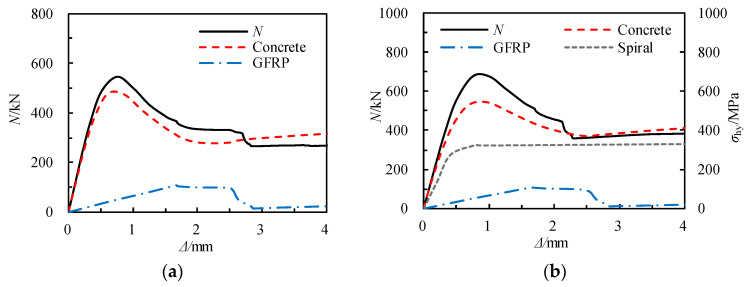
Analysis of internal forces during the stress process. (**a**) PS-C1. (**b**) PS-RC2.

Figure 16 illustrates the internal force components of specimens PS-C1 and PS-RC2 during the loading process. The initial decline in the curve is attributed to the crushing of the concrete. The location of the GFRP square tube fracture aligns with the sudden load reduction in the specimens. Compared to specimen PS-C1, specimen PS-RC2, due to the insertion of spiral reinforcement, effectively constrains the concrete in the core area, resulting in a greater load-bearing capacity and a more gradual decline in the load–displacement curve, thereby exhibiting superior ductility.

## 6. Parametric Analysis

To deeply understand the influence of key design parameters on the axial compressive behavior of RCFSGT columns, the three parameters of GFRP square—tube wall thickness t, spiral reinforcement diameter *d*_hy_, and spiral reinforcement yield strength *f*_hy_—were extended based on the verified finite element model PS-RC2.

### 6.1. Wall Thickness of GFRP Square Tube

**Figure 17:**
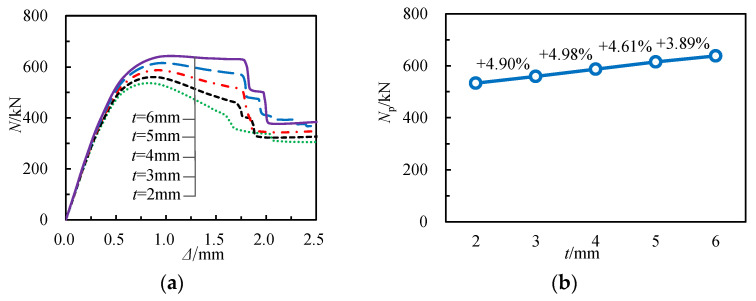
Influence of wall thickness of GFRP square tube: (**a**) load–displacement curves, (**b**) bearing capacities.

To investigate the influence of GFRP square tube wall thickness t on the load–displacement (*N*-Δ) curve and axial compressive capacity of specimens, five specimens with wall thicknesses of 2, 3, 4, 5, and 6 mm were analyzed using the finite element model. As evident from Figure 17, when compared to the 2 mm thick specimen, the bearing capacities of those with 3, 4, 5, and 6 mm thick walls increased by 4.90%, 10.13%, 15.20%, and 19.69%, respectively. Nevertheless, due to the brittleness of GFRP square tubes, there is still a sudden decrease in load with increasing *t*. The findings indicate that as t increases, the initial slope of the *N*-Δ curve rises modestly, resulting in an increase in bearing capacity but a decrease in deformability.

### 6.2. Stirrup Ratio of Spiral Reinforcement

**Figure 18:**
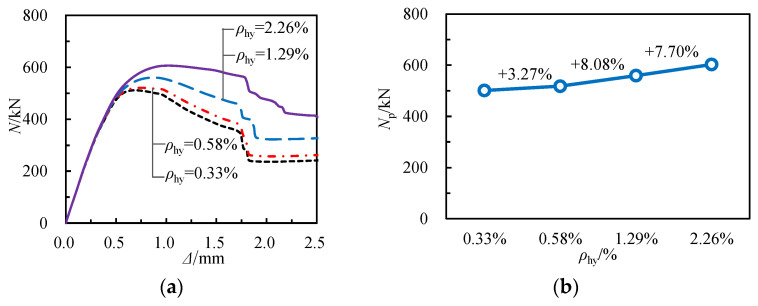
Influence of stirrup ratio of spiral reinforcement: (**a**) load–displacement curves, (**b**) bearing capacities.

Figure 18 illustrates the influence of varying spiral reinforcement stirrup ratios on the *N*-Δ curve and axial compressive capacity of specimens. The data in Figure 18 indicate that as the stirrup ratio of spiral reinforcement increases, the initial slope of the *N*-Δ curve remains constant while the load-bearing capacity is significantly enhanced. Specifically, when the stirrup ratio of spiral reinforcement rises from 0.33% to 2.26%, the load-bearing capacity increases from 501.33 kN to 602.61 kN, a rise of 20.21%. Compared to specimens with a stirrup ratio of 0.33% for spiral reinforcement, the peak displacement rises from 0.58 mm to 0.91 mm when the stirrup ratio for spiral reinforcement increases to 2.26%, marking an increase of 56.90%. This suggests that enhancing the stirrup ratio for spiral reinforcement positively influences the deformation capacity of RCFSGT columns.

### 6.3. Yield Strength of Spiral Reinforcement

**Figure 19:**
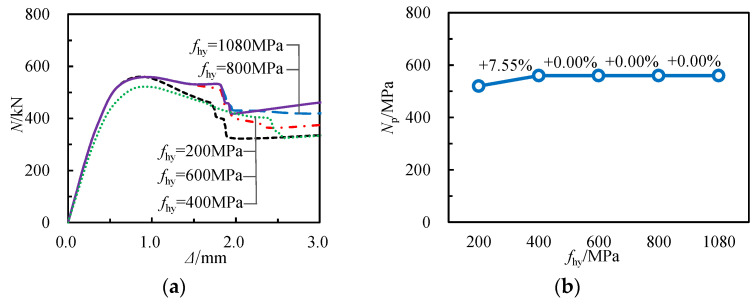
Influence of yield strength of spiral reinforcement: (**a**) load–displacement curves, (**b**) bearing capacities.

Figure 19 illustrates the influence of varying yield strengths of spiral reinforcement (*f*_hy_) on the *N*-Δ curve and axial compressive capacity of specimens. The data indicate that as FHY rises from 200 MPa to 400 MPa, the bearing capacity of the specimens increases from 520.28 kN to 559.56 kN, a rise of 7.55%. However, as *f*_hy_ continues to increase, there is minimal change in the bearing capacity. This suggests that within the range of 400 MPa to 1080 MPa, the yield strength of spiral reinforcement has minimal influence on the axial compressive capacity of specimens.

## 7. Conclusions

This study presents experimental research on RCFSGT columns under axial compression, exploring the impact of GFRP square tube wall thickness, stirrup ratio of spiral reinforcement, and yield strength of spiral reinforcement on the axial compression performance of specimens. A detailed finite element model is introduced, enabling comprehensive mechanism analysis and parametric research. The following conclusions are drawn:

(1) The ultimate failure modes of various specimens vary, among which, the thin-walled GFRP hollow tube experiences instability failure, the thick-walled GFRP tube overall failure, the thin-walled CFSGT column spalling failure, the thin-walled RCFSGT column and reinforced concrete column shear failure, and the thick-walled RCFSGT column experiences GFRP tube bulging failure.

(2) Increasing the wall thickness of the GFRP square tube significantly enhances the ultimate bearing capacity of RCFSGT columns, but reduces their deformation capacity.

(3) Increasing the stirrup ratio of spiral reinforcement is conducive to enhancing the restraint capacity of concrete and improving the bearing capacity of specimens. When the spacing of spiral bars is closer, the effect of improving the yield strength of spiral bars on the ductility of specimens is more significant.

(4) The finite element model can effectively simulate the axial loading on RCFSGT columns, demonstrating a high level of precision. The incorporated spiral reinforcement prevents the core concrete from undergoing concentrated failure in the middle, resulting in a concentration of stress at the corners of the GFRP square tube.

(5) As the wall thickness of the GFRP square tube increases, the initial slope of the *N*-Δ curve experiences a modest rise, resulting in an enhanced bearing capacity but reduced deformability. When the yield strength of the spiral reinforcement rises from 200 MPa to 400 MPa, the bearing capacity of the specimen experiences an increase of 7.55%. Further increases show minimal changes in bearing capacity. As the stirrup ratio of the spiral reinforcement continues to rise, the initial slope of the *N*-Δ curve remains constant, leading to a significant increase in bearing capacity and a commendable improvement in deformability.

(6) In practical marine engineering applications, the use of RCFSGT columns is recommended. The GFRP tube is recommended to be thin-walled, and the spiral reinforcement should be high-strength steel. The reinforcement ratio between spiral reinforcement and longitudinal reinforcement should meet the structural requirements.

## Figures and Tables

**Table 1:** Parameter design and key experimental results.

Specimen	*t*/mm	*S*/mm	*f*_hy_/MPa	*n*	*N*_p_/kN	∆_y_/mm	∆_u_/mm	μ
PS-F1	3	-	-	-	292.0	2.92	2.56	0.88
PS-F2	6	-	-	-	65.4	1.18	1.71	1.44
RC3	-	15	300	4	490.0	1.50	1.77	1.18
RC4	-	15	1200	4	509.2	1.86	11.88	6.39
PS-C1	3	-	-	-	508.0	1.42	1.54	1.08
PS-RC1	3	22	300	4	509.3	1.62	2.15	1.32
PS-RC2	3	15	300	4	485.6	1.67	2.42	1.45
PS-RC3	3	10	300	4	548.8	1.93	3.10	1.61
PS-RC4	3	22	1200	4	575.2	1.76	3.88	2.21
PS-RC5	3	15	1200	4	602.4	1.65	10.82	6.55
PS-RC6	3	10	1200	4	593.1	2.31	18.86	8.17
PS-RC7	6	22	300	4	664.3	2.59	2.87	1.11
PS-RC8	6	15	300	4	650.2	2.58	3.02	1.17
PS-RC9	6	10	300	4	769.5	2.89	3.40	1.18
PS-RC10	6	22	1200	4	819.2	2.80	3.24	1.16
PS-RC11	6	15	1200	4	930.8	2.60	3.07	1.18
PS-RC12	6	10	1200	4	926.7	2.91	3.24	1.11

**Table 2:** Mechanical properties of GFRP tube materials.

Type	Axial Tensile Strength/MPa	Transverse Tensile Strength /MPa	Axial Compressive Strength/MPa	Transverse Compressive Strength/MPa	Axial Compression Modulus/GPa	Axial Tensile Modulus/GPa
*t* = 3 mm	488.9	24.7	74.7	30.3	64.0	17.3
*t* = 6 mm	439.1	17.8	158.7	34.9	37.7	12.3

**Table 3:** Mechanical properties of steel.

Type of Steel	Yield Strength/MPa	Tensile Strength/MPa	Fracture Strain/%	Elastic Modulus/GPa
Galvanized steel wire	239	321	26.9	137
High-tensile steel wire	1419	1567	2.0	157

**Table 4:** Summary of experimental and FE results.

Mark	N_u,EXP_ (kN)	N_u,FEA_ (kN)	N_u,EXP_/N_u,FEA_
PS-RC1	509.28	497.29	1.02
PS-RC2	485.55	520.28	0.93
PS-RC3	548.82	551.52	1.00
PS-RC4	575.22	618.86	0.93
PS-RC5	602.37	676.79	0.89
PS-RC6	618.96	742.31	0.83
PS-RC7	664.32	658.96	1.01
PS-RC8	650.16	624.89	1.04
PS-RC9	769.47	713.17	1.08
PS-RC10	819.21	800.00	1.02
PS-RC11	930.81	820.25	1.13
PS-RC12	926.67	787.33	1.18
Average			1.01
Standard deviation			0.09

## Data Availability

Data are contained within the article.
